# Assisted Cycle Therapy (ACT) Improved Self-Efficacy and Exercise Perception in Middle-Age Adults with Down Syndrome

**DOI:** 10.3390/brainsci13121719

**Published:** 2023-12-15

**Authors:** Shannon D. R. Ringenbach, Nathaniel E. Arnold, Kori Tucker, Miya K. Rand, Breanna E. Studenka, Stockton B. Ringenbach, Chih-Chia Chen

**Affiliations:** 1 College of Health Solutions, Arizona State University, Phoenix, AZ 85004, USA; nearnol1@asu.edu (N.E.A.); kori.tucker.51@gmail.com (K.T.); rand@asu.edu (M.K.R.); sbringen@asu.edu (S.B.R.); 2 Department of Kinesiology and Health Science, Utah State University, Logan, UT 84322, USA; breanna.studenka@usu.edu; 3 Department of Kinesiology, Mississippi State University, Starkville, MS 39762, USA; cc2196@msstate.edu

**Keywords:** physical activity, intellectual disability, mental health, Alzheimer’s disease, rehabilitation

## Abstract

Alzheimer’s disease is prevalent in persons with Down syndrome (DS) as early as their 30s and presents as decreased social interaction, coordination, and physical activity. Therefore, changing attitudes and beliefs about exercise is key to increasing motivation for physical activity especially in middle-age adults with DS. The aim of this study was to examine the effects of Assisted Cycle Therapy (ACT) on self-efficacy and exercise perception in middle-age adults with Down syndrome (DS) following an exercise intervention three times a week for 8 weeks. Twelve participants were in the ACT group in which a motor assisted their cycling to be performed at least 30% faster than voluntary cycling (VC), 10 participants were in the voluntary cycling group, and two participants were in the no cycling (NC) group. The results showed that both exercise groups (i.e., ACT and VC) improved in their self-efficacy after the 8-week intervention. In addition, exercise perception improved following ACT, but not VC or NC. Our results are discussed with respect to their future implications for exercise in the DS population. The results can be attributed to differences in effort required by each intervention group as well as the neurotrophic factors that occur when muscle contractions create synaptic connections resulting in improvement in cognition and feelings of satisfaction.

## 1. Introduction

The cardinal characteristics of persons with Down syndrome (DS) typically include physical factors such as joint hypermobility, hypotonia [1], low VO_2_max [2], chronotropic incompetence [3], low exercise tolerance [4], and mental health and cognitive factors such as depression and lower IQ [5]. Cognitive deficits are the cornerstone of persons with DS and have been associated with smaller brain volumes in the cerebellum, hippocampus, frontal and temporal areas [6].

These physical and mental characteristics of individuals with DS play a role in their reduced levels of physical activity. It is suggested that all adults should be engaging in 150 min of moderate to high intensity exercise every week to gain health benefits from exercise [7]. Concerning the DS population, who already possesses a physical and mental propensity towards sedentary activity, this is a particularly relevant situation [8]. A study conducted involving adolescents found that only 58% of teens with DS are meeting the proper exercise standards; this is relatively low compared to the typical population of adolescents in which 75–85% are meeting these recommendations [9,10]. To our knowledge, there is no data concerning the amount of physical activity in middle-age adults with DS, although it is reasonable to assume that it is lower than in adolescents with DS due to the common decline in physical and mental activity and negative health consequences that are a result of physical inactivity. One of the many chronic diseases to have a high prevalence in sedentary adults is cardiovascular disease. It has been found that a dose-response relationship exists between high amounts of time spent doing sedentary activities (e.g., playing video games) and mortality from cardiovascular disease [11]. Furthermore, individuals with DS have an increased incidence of Alzheimer’s disease (AD) as they age. This increased prevalence appears to be accelerated in populations with DS by almost 30 to 40 years [12]. It is estimated that 10–25% of adults with DS have AD at age 40–49 and 20–50% have AD at age 50–59. Additionally, autopsy studies show that by age 40, the brains of almost all individuals with DS have significant levels of plaques and tangles, and abnormal protein deposits which are considered hallmarks of AD [13]. Therefore, it is extremely important that more research be done on methods of motivation and maintenance of physical activity in middle-age adults with DS.

AD presents itself differently in adults with DS and can manifest in several ways: decreased social interactions, decreased cooperativeness, change in coordination, decrease in physical activity, and changes in gait. Thus, we believe that studying the influence of an exercise program on self-efficacy and exercise perception is crucial. Changing attitudes and beliefs about exercise is key to increasing motivation for physical activity, improving cooperativeness, and enhancing social interactions. In a study by Heller and colleagues [14], 53 adults with DS completed a physical activity regimen for three days a week for twelve weeks. The most common inhibitors of exercise were found to be lack of energy, finding exercise to be boring or difficult, feelings of laziness, or having health concerns related to physical activity. The intervention group yielded significant positive changes in their perception of exercise and attitudes about getting physically active. This included increased self-efficacy, more positive-expected outcomes, fewer cognitive-emotional barriers, improved life satisfaction, and marginally decreased depression [14]. In the typical population, self-efficacy is a strong predictor of engagement in exercise [14,15,16]. 

Self-efficacy is defined by a person’s confidence in their ability to perform a task or skill successfully [15]. Individuals possessing a high self-efficacy for exercise are more likely to become and remain active throughout their life. If one believes that they can engage in or complete an exercise skill, they are more likely to do so. When an individual’s self-efficacy is initially low for physical activity, having positive experiences during exercise can consequently increase their confidence in their ability to exercise in the future, thus increasing their self-efficacy [15]. It has been found that when an individual has high self-efficacy for a task, they will attribute their failures to lack of effort and believe that they will succeed if they put in more effort on the next attempt [15]. Equally, those with lower self-efficacy for a task will attribute failures to their perceived inability to perform the skill. This causes a loss in motivation and a tendency to give up more easily [15]. 

One’s self-efficacy has more to do with their perception of their capabilities and less to do with the reality of their competency [15]. Furthermore, an increase of positive experiences in exercise will most often increase self-efficacy for that task. In contrast, if the individual’s initial self-efficacy was low for that task and negative experiences occur, self-efficacy will often decrease [15]. Because of the linear relationship between type of experience and self-efficacy, giving individuals positive experiences will prospectively increase their self-efficacy for that experience in the future, while providing negative experiences will probably yield a decrease in an individual’s self-efficacy for that task, thus confirming their initially low competency [15,17,18]. Self-efficacy has been suggested as a means for the anxiety reducing and anti-depressive factors of exercise [19,20,21]. However, the evidence that self-efficacy is the mechanism in which exercise is associated with depression and anxiety is currently correlational. More research would be required to provide a more concrete supporting argument for typical and atypical populations [19,20,21]. 

Wood & Bandura [22] conducted a study on motivation and self-efficacy by testing participants in two different mindsets. In the “fixed” mindset group, the participants were informed that they would perform a task and that their score was a direct reflection of their abilities to succeed in that specific type of test. In the “growth” mindset group, the participants were informed that for the same test, they would be assessed on “an acquirable skill” [22]. Those who were told they could acquire the skill performed much better, had a higher self-efficacy score, and set more difficult goals for performing the skill than those in the fixed mindset group. This might demonstrate that people will put in more effort and yield better results in a task when they believe that they have the capacity for improvement [22,23]. This relates to middle-age adults with DS because they may be more apt to participate in physical activity if they believe that they can perform the tasks involved successfully despite their physical and mental limitations. Accommodations such as assisted exercise can be made to make exercise tasks more approachable for middle-age adults with DS. Assisted Cycling Therapy (ACT) is completed on a stationary exercise bicycle in which a motor assists in moving the pedals at a faster rate than they can on their own and is one way that may motivate middle-age adults with DS in believing a task, such as cycling or any other exercise skill, can be achievable. This belief may increase an individual’s self-efficacy and enthusiasm about exercise. 

In other populations, such as Parkinson’s disease (PD), assisted exercise (AE) has shown promising results [24,25,26] and has been recently applied to a population of adolescents with DS [27]. AE requires a participant to exercise at a higher rate than they would during voluntary exercise (VE) usually through the use of a motor or other mechanism that increases either speed, resistance, or intensity. VE represents the pace or intensity that the participant would naturally select [25]. AE has shown improved neuroprotective properties, motor function, and increased cerebral blood flow in participants with PD. These factors improved performance in cognitive skills and had a trend in decreasing depression [24,25,26]. Specifically, ACT is a form of AE which utilizes a stationary bicycle with a motor. During ACT, an individual will pedal on the bike and the motor will increase the RPM automatically, thus allowing faster pedaling with no extra effort [27]. This study demonstrated that ACT may be a promising method of AE in the DS population. The 2014 study utilized an acute ACT intervention that analyzed whether participants with DS could improve their motor control, cognition, exercise perception, and self-efficacy after one bout of 30-min exercise of ACT. The results demonstrated that information processing, cognitive planning, and exercise perception were all improved following one session of ACT but not following Voluntary Cycling (VC) on the stationary bike or no exercise at all. Self-efficacy did not change significantly after one session; however, this is not to say that a more chronic regimen might yield different results. In fact, it was later discovered that SE could be improved following ACT after an 8-week intervention in adolescents with DS [28].

To our knowledge, no studies have utilized a long-term (i.e., 8-week) between-group intervention using ACT in middle-age adults with DS and measured changes in self-efficacy and exercise perception. Thus, this paper aims to further analyze the effects of ACT and voluntary cycling (VC) on self-efficacy as well as exercise perception, assessed using subsets of the Physical Activity and Self Efficacy Survey [29], in middle-age adults with DS. Based on preliminary data from our lab with adolescents with DS [28], it was hypothesized that ACT would improve self-efficacy and exercise perception after 8 weeks, more so than VC, and no improvements were predicted following the no cycling (NC).

## 2. Materials and Methods

### 2.1. Participants

As can be seen in Table 1, participants consisted of 24 adults with Down syndrome (DS), with a mean age of 36.4 years and a mean mental age of 6.1 years. Participants were recruited through flyers, word of mouth in the community, phone call connections, and email announcements. First, parents or legal guardians completed a “Physical Activity Readiness Questionnaire” on behalf of the participant where seven answers of “no” determined the participant’s eligibility and readiness for exercise. All protocols used in this intervention were approved by the Human Subjects Institutional Review Board of Arizona State University.

Twelve participants were randomized into the Assisted Cycle Therapy (ACT) group, ten were randomized into the Voluntary Cycling (VC) group, and two were placed in the No Cycling (NC) control group via convenience sampling.

### 2.2. Exercise Interventions

Participants in both exercise groups participated in a supervised exercise protocol for eight weeks, for at least three 30-min sessions per week. Exercise intensity, from an aerobic perspective, was matched for both exercise groups. Intensity was determined on an individual patient basis based on the formula specific for persons with intellectual disabilities. Fernhall and colleagues [30] developed a formula for predicting maximal HR for persons with DS (i.e., MaxHR = 210 − (0.56 × age) − (15.5 × DS)), with DS coded as 2. Training heart rate (T_hr_) zone for each participant, based on ACSM recommendations of exercise management for persons with chronic diseases and disabilities, was 60–80% peak HR range [31]. The participants were instructed to exercise within their T_hr_ during the 30-min main exercise set. The main exercise set occurred after a 5-min warm-up phase. 

### 2.3. Apparatus

Participants utilized a stationary recumbent bicycle that contained a mechanical motor that would assist them in pedaling at a preset rate during each exercise intervention. The pedals included strap and cage devices to hold the participants’ feet in place for comfort. Participants wore athletic shoes and clothing for all interventions. For each intervention, the participants were asked to sit on the bike and pedal either voluntarily or to the motor set pace. The pedaling cadence was set by a CycleOps Cervo 2.4 cycle computer that was wired to a magnetic cadence sensor hooked up to the bike. Participants wore Bontrager ANT+ Softstrap Heart Rate belts that synchronized with the cycling sensor computer to determine Heart Rate. 

### 2.4. Experimental Groups

1. Assisted Cycle Therapy (ACT): Training pedaling rate was set at 35 percent greater than the participant’s preferred pedaling rate, determined during the warm-up of each exercise session. A 35% increase is consistent with previous research using this methodology [26,27]. During exercise, the participant’s HR was displayed relative to their THR zone on the cycle monitor. Participants were instructed to maintain their HR within their THR zone through active pedaling of the cycle and adjust (increase or decrease) their contribution to the pedaling action in order to maintain their HR within THR. The algorithm controlling the motor is responsive to pedaling rate, patient work, and HR; these data were used to determine the level of assistance or resistance provided by the motor. 

2. Voluntary Cycling (VC): The exercise cycle was operated in its standard mode in which the motor does not provide any assistance with pedaling. Participants were instructed to maintain HR within their individualized T_HR_ zone. No instructions were given regarding the maintenance of a particular cadence. Cadence and resistance level were voluntarily selected by the participant. The researcher ensured the participants maintained their HR within T_HR_ during the main exercise set. 

3. No Cycling (NC) Control Group: Participants in the no-exercise group were asked to maintain their current level of activity throughout the study period. 

### 2.5. Dependent Measures

Self-Efficacy and Exercise Perception Questionnaires: Participants completed two questionnaires at the beginning and end of their 8-week session. These questionnaires were designed to evaluate their beliefs in their ability to perform exercise and how exercise could help them. 

#### 2.5.1. Self-Efficacy Questionnaire

This questionnaire was specially developed for individuals with intellectual disabilities and it is a subdivision of the Physical Activity and Self-Efficacy Survey [29]. The purpose of the survey is to evaluate the participant’s belief in his or her ability to perform the exercise protocol for the given intervention time, despite varying circumstances. Participants were asked to respond “yes”, “no”, or “maybe” to the following questions: “Do you think you can make time for this activity almost everyday?”, “Do you think you can do this activity even when you are very busy?”, “Do you think you can do this activity even when you are feeling sad?”, “Do you think you can do this activity even after a long, hard day at school?”, “Do you think you can do this activity on days when you are tired?”, “Do you think you can do this activity when you feel lazy?”. “Yes” was scored as a 3, “maybe” was scored as 2, and “no” was scored as 1. The higher the individual’s score, the more the participant believed in his or her ability to bicycle every day.

#### 2.5.2. Exercise Perception

This questionnaire measured the participant’s perception of how exercise would affect him or her. It was a part of the Physical Activity and Self Efficacy Survey that was developed specifically for individuals with an intellectual disability [29]. Participants were asked if exercise would “help”, “not help”, or “neither or both” to the following conditions: lose or control their weight, make them feel tired, make them happier, make them hurt less, help them to meet new people, help them to get in shape, make them look better, and improve their health. “Help” was scored as a 3, “neither or both” was a 2, and “not help” was 1. The higher the individual’s score, the higher the participant’s belief that exercise would help them. 

### 2.6. Data Analysis

The ACT, VC, and NC groups were analyzed independently to look for differences in data from pretest to posttest. Separate one-tailed paired sample *t*-tests were conducted for each dependent measure (i.e., self-efficacy, exercise perception) pre and post intervention with SPSS version 21. 

## 3. Results

### 3.1. Self-Efficacy

**Figure 1:**
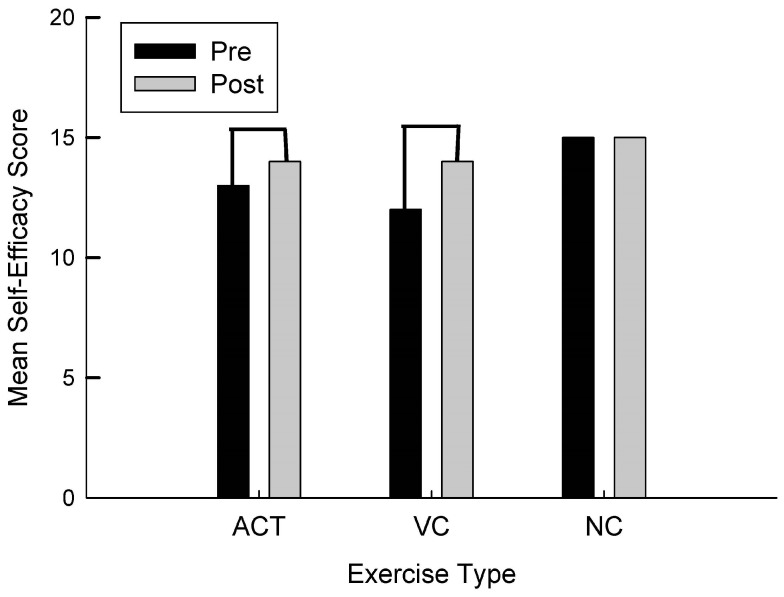
Mean self-efficacy score as a function of group (ACT, VC, NC) and time (pre, post).

For self-efficacy, there was a significant difference in the VC intervention t(9) = −3.25, *p* = 0.005 in which self-efficacy improved from pre to post. As can be seen in Figure 1, For the ACT intervention, there was a trend toward conventional levels of significance t(11) = −1.40, *p* = 0.095 in which self-efficacy improved from pre to post. There were no differences in the NC intervention.

### 3.2. Exercise Perception

**Figure 2:**
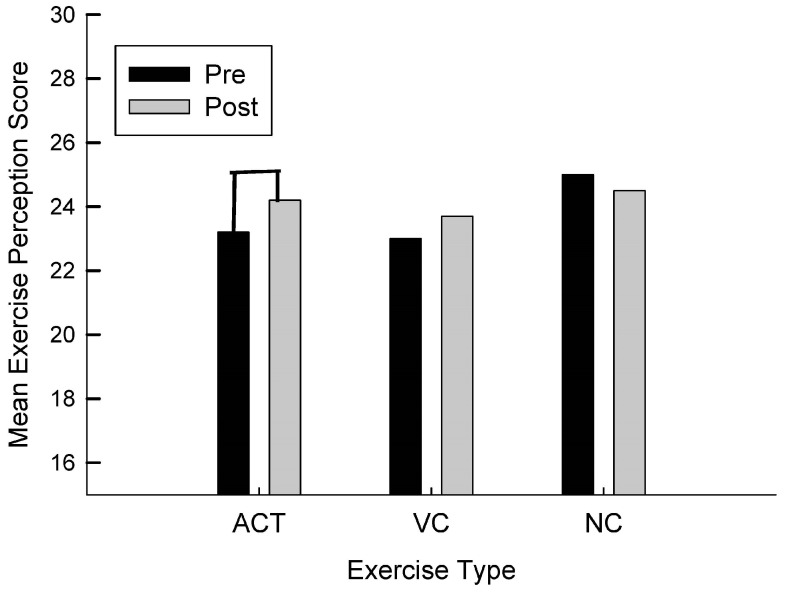
Mean exercise perception score as a function of group (ACT, VC, NC) and time (pre, post).

For exercise perception, as can be seen in Figure 2, there was a trend towards conventional levels of significance for the ACT intervention t(11) = −1.66, *p* = 0.063 in which exercise perception improved from pre to post. While the VC group improved slightly, it was not significant t(9) = −0.94, *p* = 0.19 and the NC group did not improve t(1) = 1.00, *p* = 0.250.

## 4. Discussion

This is the first study, to our knowledge, that has utilized a long-term (i.e., eight-week) between-group intervention using Assisted Cycling Therapy (ACT) in middle-age adults with DS and measured changes in self-efficacy and exercise perception as measured by the PASE survey [29]. This is important because when people with Down syndrome are more confident and believe that exercise is beneficial, they will be more physically active which will ultimately improve their overall quality of life. 

### 4.1. Self-Efficacy

Our results are somewhat consistent with our hypotheses that the ACT and VC groups improved their self-efficacy after an 8-week exercise intervention. Our results showed that this was significant in the VC group and approached conventional levels of significance in the ACT group. The ACT results, however, are consistent with preliminary data from our lab on adolescents with DS [28]. In our previous study, there were 39 adolescents with DS between the ages of 9 and 26, and they found more improvements in self-efficacy following ACT than VC; however, in our study we found larger differences following VC than ACT. Similarly, a pilot study with seven middle-age adults with DS in our lab found that self-efficacy improved following an 8-week intervention with ACT, but did not improve following VC or NC [32]. These results are also consistent with a study investigating self-esteem following 60 min of a supervised walking program 3x/week, 90 min of yoga 2x/week, or no exercise in low-active older women which found that both exercise interventions increased the subdomain self-esteem, which after 12 weeks improved global self-esteem and self-efficacy as it relates to physical activity and body attractiveness [33]. The Elavsky study demonstrated how an exercise intervention could positively affect the physical self-esteem of typical low-active older women. In a study with 174 typical older adults (Mage = 66.7 years), there was indirect support for physical activity to increase self-efficacy on the physical self-worth and global esteem through the subdomain of esteem. Furthermore, these positive correlations continued over a 4 year period [34]. 

Unlike in the hypothesis, it was found that VC produced a greater increase in self-efficacy than ACT. One explanation is that when a participant is putting in physical work, there is effort and energy invested in the process so their belief in their abilities to be physically active are higher than an ACT participant who had much of the work done for them by the bike motor. A study conducted by David and colleagues [35] measured the overall trait self-efficacy, daily state self-efficacy, and daily pedometer measurement from 71 post-menopausal women over 84 days and found that state self-efficacy was a robust predictor of future physical activity regardless of trait self-efficacy and total steps. This demonstrates the direct relationship that exerting voluntary effort can contribute to one’s overall feelings of self-efficacy at the end of a bout of exercise. 

### 4.2. Mechanisms of ACT

**Figure 3:**
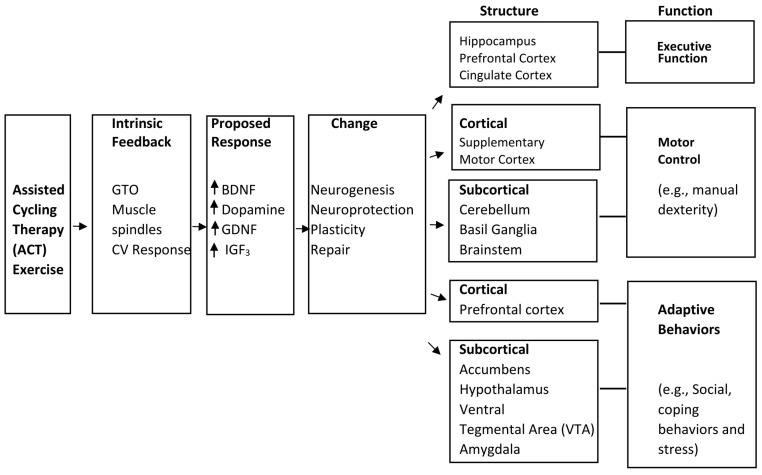
Model of structure and function responsible for ACT and changes in behaviors.

Neural plasticity and repair and their effects on cognitive and motor behaviors have been studied as they relate to ACT exercise. In a study conducted by Alberts and colleagues [24], Parkinson’s patients were found to pedal at a faster and a more steady pace through ACT than through VC. This increased the quality and amount of intrinsic neural feedback from the muscles to the brain causing neurotrophic factors BDNF, GDNF, and IGF3 to be released in the brain. These factors relate to neural plasticity and neurogenesis, both of which would further synaptic connections, thus training muscle habits in participants giving us the belief that improvements in cognition are possible. As can be seen in Figure 3, it has been suggested that these factors of neural plasticity and neurogenesis may increase learning memory, motor control, and behavioral adaptations [24]. By creating more nervous tissue and nervous tissue connections, the brain is able to operate at an increased level of connection. 

These connections that are made reach the amygdala and the prefrontal cortex (PFC) in the brain which are predominately responsible for emotion [36]. Consequently, in the correlation of neurotrophic factors and muscle nerve connection due to assisted cycling, these factors improve emotion by constructing developments on the amygdala and PFC [24]. Thus, feelings associated with depression and anxiety may be reduced thus resulting in an increased positive affect and overall emotions of satisfaction or affirmation [24,36]. Positive affect has been found to be related to increased self-efficacy [15,17,37]. If the neurotrophic factors discussed in relation to muscle contractions during ACT and VC are acting on the emotion and affect centers of the brain, this mechanism of increasing positive affect could explain our resulting improvements in self-efficacy following exercise intervention [15,24].

### 4.3. Exercise Perception

As previously discussed, the ACT intervention yielded conventional significance for an increase in exercise perception while VC improved with no significance and NC yielded no improvements. It is possible that because the participants in the ACT intervention were pedaling at such a high cadence from the motor, this caused them to feel as if they were innately better at exercising. It also caused less fatigue because of the lack of effort in comparison to those participants who had to voluntarily pedal in the VC group. Exercise perception improves when one feels that they have feasible accessibility, enough energy to complete activities, and positive motivation to do so. A study conducted by Sowers and colleagues [38] measuring the exercise perception and behaviors of individuals with primary immunodeficiency disease (PID) found that participants found exercise to be feasible and better for mental and physical well-being post intervention. Highly functioning participants possessed the perception that exercise could not worsen their health or mental conditions but only improve them. One explanation as to why none of the intervention groups reached significance for this variable could be that the participants came in with a generally positive attitude and tend to have a positive outlook on opportunities and situations in other areas of their lives already. This mindset most likely caused high initial scorings on exercise perception as well as a possible lack of understanding of what exercise entails and its overall benefits. The positive encouragement, consistency of interaction with researchers, and community socialization aspects of the intervention could have caused the improvements in exercise perception that were found post intervention, however, these improvements were not statistically significant. 

A possible explanation for lesser improvements in VC than ACT for exercise perception is that the task might have felt too difficult, making exercise feel less feasible. ACT participants had to put in minimal effort, making exercise seem more “doable” and producing an overall more positive outlook on physical activity while VC participants had to put on all of the work making their physical ability obstacles more demanding to overcome. This challenge could be the reason that the VC participants reported lower exercise perception than ACT participants. Previous research has shown that it is common in more sedentary individuals for their exercise perception and even self-efficacy to lower after a difficult bout of exercise of high intensity or longer amount of time [17]. In a study conducted by Woo and colleagues [18], college students with no intellectual disabilities participated in low, moderate, and high intensities of exercise protocols. The study measured resulting positive affect for all exercises compared to baseline. The positive affect was measured by comparing activation levels of the left and right frontal hemispheres of the brain. Positive affect as measured by left frontal activity has been found to be correlated with greater motivation to be physically active [39,40], therefore when the left frontal hemisphere is greater in activation compared to the right, more feelings of motivation (associated with the left hemisphere) are present as opposed to avoidance or negativity (associated with the right hemisphere) [18]. As previously discussed with self-efficacy, the overall activation of the frontal cortex has shown to improve positive affect, thus improving both exercise perception associated with the intervention and overall self-efficacy [15,17,37]. 

Another approach suggests that improvements in self-efficacy for certain tasks could also play a role in the improvement of exercise perception. This correlation proposes that positive experiences with exercise might not only increase one’s confidence in their abilities for that skill (SE), but also help them to perceive the physical task as something doable and even possibly enjoyable (EP). Self-efficacy is a suggested mechanism for contribution to the anti-depressive and anxiolytic effects of exercise, thus supporting the idea of this reverse effect connection [19,20,21]. A suggested hypothesis could be that an increase in SE after ACT and VC was not only caused by new brain synaptic connections due to neurotrophic factors, but also by continuing to gain positive experiences during intervention. These positive experiences could have been formed by the encouraging environment created by the researchers. It has been found that people will have higher self-efficacy and perform better on a task if they are told that the skill is “acquirable” instead of “innate”. Having researchers involved in the sessions encouraging participants to work their hardest and commending their improvements likely increased their self-efficacy for cycling as well as their perception of exercise itself. This overall experience, combining positive encouragement, improvement, and motivation is analogous to telling participants that exercise is “acquirable” by giving them the skills to feel that progress is attainable and can even be enjoyable. Researchers provided the same encouragement and support for all participants, regardless of intervention group. This consistency supports the idea that the encouragement is not a confounding factor that influenced the participants’ self-efficacy. If it were a factor, self-efficacy and exercise perception would have improved similarly across ACT and VC following intervention, however this was not the case. 

It has been discovered that sedentary individual’s affect during exercise can predict their efficacy post-exercise and thus give an indication of their overall likelihood to return to exercise in the future (exercise perception) [37]. Each time participants came into the intervention lab, their predisposed perception and overall efficacy for the task pre-intervention was most likely based off of how they felt during and after intervention the previous time. As this intervention was 8 weeks long, it gave participants the privilege of expectations for sessions and consistency of growth. If they had positive experiences, regardless of intervention, it is logical that they will come in expecting their experience to be similar to before, thus increasing exercise perception. Low levels of physical exhaustion during exercise have been found to be associated with more positive affect produced during exercise, thus yielding higher levels of post-exercise self-efficacy and future perception of exercise itself as it pertains to specific tasks [17]. This even further relates to our gap in improvement from ACT to VC because the participants in ACT were less physically exhausted than participants in VC, making it reasonable that their perception of exercise improved slightly more than the VC intervention group. 

### 4.4. Exercise Intensity

It is important to acknowledge that overall, the difficulty and intensity of exercise had an effect on the participants’ self-efficacy and exercise perception post exercise. A study conducted in our lab by Chen and colleagues [41] researched how 20 min of treadmill affected different groups with Intellectual disabilities (IDs) including one group of individuals with DS. The participants rated their exhaustion and contrary to typical people without ID, the results displayed that populations with ID did not experience the same level of positive affect following exercise due to the length of time and intensity increase throughout the exercise. This could be because the exercise was too challenging or physically demanding to experience increased positive affect, as it has been found that individuals with IDs can have an increased positive affect after exercise as long as the bout is not too high of a difficulty [18]. Other studies have demonstrated that positive affect can be attained from exercise in individuals with IDs as long as they are exercising below ventilatory threshold (VT) which in this population tends to be about 60–70% of their VO_2_ max [42]. Because the VC intervention group was pushing themselves to pedal and work hard, they tended to become more physically fatigued during exercise than the ACT group who did not have to output much energy due to the motor. As the VC participants experienced exhaustion, this created negative affect in the brain which could potentially result in a more negative experience for those individuals and therefore less of an increase in exercise perception than in ACT. Overall, there seems to be a trend in the research identifying intensity and duration of exercise as the most prominent variable involved with positive affect fluctuation during and after exercise. 

It has been established that low to moderate intensity of exercise increases affect in individuals with IDs, and positive affect predicts more positive exercise perception, thus, this makes ACT a valuable and reliable option for persons with Down syndrome [15,17,18,37,41,42]. This light intensity solution can continue to improve overall self-efficacy and exercise perception in individuals with DS and other intellectually disabled individuals. This approach could be used in future studies to determine if there are similar affects in individuals with other IDs or other chronic illnesses. ACT could also be used as an alternate track for sedentary individuals or already obese individuals to introduce exercise as a positive and beneficial experience. Having participants who have negative past experiences with exercise and low confidence to exercise could benefit from the encouragement and the lack of effort required to participate in ACT. This may not be a viable solution for overall lifetime fitness; however, it is a way to build personal self-efficacy for exercise and to hopefully create new positive experiences that will develop an overall more positive exercise perception that encourages more motivation within any individual who participates. 

ACT is a universal exercise that can be implemented in a plethora of populations, ages and ability levels. In the future, aside from solely changing the population, other factors can be included to improve the overall experience. Previous studies have shown that social accountability and peer interaction can improve motivation and overall experience with exercise. Barr & Shields [43] conducted a study with adolescents with Down syndrome that found that a major contributing factor to their physical activity was engaging in positive peer interaction during their exercise. A parent from this study shared that this type of comradery has been shown to encourage her son in the past and is very common within the DS population. This psychological component as well as other motivating factors could be added into the program to hopefully heighten self-efficacy and overall perception of health and physical activity. It is necessary to make adaptations to fit the specific needs of those with IDs when it comes to exercise because without them, the individuals will lose interest and not be consistent enough or even at all to gain the health benefits of exercise. Improving self-efficacy and exercise perception will not only introduce many opportunities for physical activity that many people may not have been aware of, but it will encourage them to be more active, more social, and lead overall healthier lifestyles. 

### 4.5. Limitations

Individuals with DS have several different levels of functionality physically, developmentally, and mentally. Within the ACT intervention group, the mental age of the participants ranged from 2.75 to 12.75 years old. This limitation caused a high level of variance upon pre/post responsiveness and overall intervention participation. These variances cannot be eliminated from the study, but this is a common occurrence in research with special populations and maybe in further research there will be a more accurate way of measuring responses. In addition, the control group was a convenience sample of participants that could not travel to do the study and while it was a small group, they had a higher chronological age and a lower mental age. For the purposes of this study, this group demonstrated that if you do nothing, you gain nothing which was important to demonstrate. However, future research should have a larger, age-matched control group.

Furthermore, survey results are self-reported causing variability in certainty and comprehension between each individual, which can limit the validity of the results. However, these surveys were designed and validated on persons with DS making them the most appropriate dependent measures at this time. 

## 5. Conclusions

The results were mostly consistent with the hypothesis that self-efficacy and exercise perception would improve more after ACT than VC and not at all after NC. For self-efficacy, ACT trended towards conventional significance while VC was statistically significant. For exercise perception, ACT trended towards conventional significance while VC was not significant. With these results, eight weeks of moderate exercise via ACT may yield higher self-efficacy and exercise perception in middle-age adults with DS. For the application of these results, it is suggested that persons with DS participate in initially easy physical activity to gain positive experience to encourage them to perceive exercise in an enjoyable manner and have the confidence in their abilities to complete exercise in the future. This will only further increase self-efficacy, and will lead these individuals to live more healthy and autonomous lives. This constant conditioning of positive association with exercise will motivate them to continue to be more active, seek out opportunities to be more active, and to believe in themselves to do so. It is important to acknowledge the overall importance that giving positive experiences and increasing the tendency to exercise for middle-age individuals with DS who are in desperate need of improving their health is a success even if it is the slightest amount. Insignificant statistical results do not mean significant positive changes did not occur in the lives of these people and we firmly believe that middle-age adults with DS benefited because of this intervention to exercise alone and to continue exercises for an overall healthier lifestyle.

## Figures and Tables

**Table 1:** Participant characteristics.

	ACT (n = 12)		VC (n = 10)		NC (n = 2)	
	Mean	SD	Mean	SD	Mean	SD
Chronological age (years)	38.0	8.81	36.2	9.27	52.9	1.88
Mental age (years)	6.9	3.09	6.3	2.66	5.59	4.59

## Data Availability

The data presented in this study are available on request from the corresponding author. The data are not publicly available due to privacy considerations.
